# A Botanical Mixture (NWG11-02) of *Elaeagnus angustifolia* L. (oleaster) and *Sophora japonica* L. Fruit Extract Alleviates Monoiodoacetate-Induced Osteoarthritis in Rats

**DOI:** 10.4014/jmb.2509.09022

**Published:** 2025-12-09

**Authors:** Seohan Kim, Seongjun Kim, Myeonghwan Oh, Jinyeong Lim, Wonchul Lim, Tae-Gyu Lim

**Affiliations:** 1 Department of Food Science & Biotechnology, Sejong University, Seoul 05006, Republic of Korea; 2 Xenbio Group Co., Ltd., Seoul 05006, Republic of Korea; 3 Life Science Research Institute, NOVAWells Co., Ltd., Cheongju, Chungbuk 28126, Republic of Korea

**Keywords:** Cartilage degradation, *Elaeagnus angustifolia*; inflammation, MIA-induced rat model, Osteoarthritis, *Sophora japonica* L.

## Abstract

Osteoarthritis (OA) is a chronic degenerative joint disease characterized by progressive cartilage degradation, synovial inflammation, and impaired joint function. This study investigated the therapeutic potential of a botanical mixture, NWG11-02, comprising *Elaeagnus angustifolia* L. (oleaster) extract (OE) and *Sophora japonica* L. fruit extract (SJFE) in a monosodium iodoacetate (MIA)-induced OA rat model. Oral administration of the mixture for four weeks significantly improved weight-bearing function and preserved cartilage architecture, as evidenced by reduced Mankin scores and enhanced proteoglycan retention in toluidine blue-stained sections. Molecular analyses revealed that the mixture markedly enhanced the expression of cartilage anabolic markers (aggrecan and type II collagen) and reduced matrix-degrading enzymes (MMP-3 and MMP-13), while suppressing the expression of pro-inflammatory cytokines (COX-2, TNF-α, IL-1β, IL-6) at both mRNA and protein levels. These effects exhibited a dose-dependent pattern, with the medium-dose mixture (80 mg/kg) demonstrating superior efficacy compared to individual extracts. No systemic toxicity was observed during the treatment period. Collectively, these findings suggest that NWG11-02 exerts multi-faceted protective effects on joint tissues and may serve as a promising natural therapeutic strategy for OA management.

## Introduction

Osteoarthritis (OA) is the most widespread form of arthritis and a major contributor to long-term disability worldwide [1]. It is characterized by progressive cartilage degradation, subchondral bone remodeling, synovial inflammation, and the formation of osteophytes [2, 3]. Clinically, OA is characterized by joint pain, stiffness, and limited mobility [4] with the knee being the most frequently involved site [5, 6]. Conventional OA treatments like NSAIDs and acetaminophen relieve pain and inflammation but may cause multiple adverse effects including gastrointestinal and cardiovascular toxicity with long-term use [7]. Surgical interventions, while effective in advanced cases, carry risks such as infection and revision surgery, especially in elderly patients [8]. These limitations have prompted growing interest in natural product-based therapies as safer alternatives. Various plant-derived materials have been actively investigated for their potential to prevent or alleviate OA-related symptoms and joint degeneration [9-12].

*Elaeagnus angustifolia* L. (oleaster) extract (OE) has traditionally been used for its antioxidant, wound-healing, and muscle relaxant effects, with studies supporting its therapeutic potential in OA [9]. Clinical trials have shown that oleaster extract alleviates pain, reduces stiffness, and improves physical function in knee OA patients, with efficacy comparable to ibuprofen [13]. In preclinical models, oleaster treatment improved cartilage integrity by enhancing antioxidant enzyme activities and decreasing malondialdehyde (MDA) levels [14]. Furthermore, alcohol extracts of oleaster, specifically prepared using absolute ethanol, have demonstrated notable anti-inflammatory effects [15]. Similarly, *Sophora japonica* L. fruit extract (SJFE), rich in flavonoids such as rutin and kaempferol, exhibits strong protective effects against osteoporosis [16], along with anti-inflammatory and potential OA-related benefits [17-20].

While both OE and SJFE have individually demonstrated therapeutic benefits, their combined effects have not been explored. Notably, previous studies have shown that combinations of plant-derived extracts, such as ginger and honey, exhibit synergistic anti-inflammatory and antioxidant effects [17]. Similarly, the combination of *Boswellia serrata* and *Apium graveolens* (celery) has been reported to enhance joint function and reduce pain in OA patients more effectively than individual treatments [21]. These observations indicate that combining oleaster and SJFE may offer enhanced joint-protective benefits.

Previous studies have shown that several molecular markers are closely associated with the progression of OA. Pro-inflammatory cytokines, including tumor necrosis factor-α (TNF-α), interleukin-1β (IL-1β), and interleukin-6 (IL-6), are known to promote synovial inflammation and cartilage degradation [22]. Cyclooxygenase-2 (COX-2) is also involved, mainly through its role in prostaglandin production and pain signaling [23]. In addition, matrix metalloproteinases such as MMP-3 and MMP-13 accelerate the breakdown of extracellular matrix components, particularly type II collagen and Aggrecan (ACAN), which are key to maintaining cartilage structure [24]. On the other hand, anabolic markers like type II collagen alpha 1 chain (COL2A1) and ACAN are important indicators of cartilage repair and matrix stability [25]. Therefore, tracking the expression patterns of these markers can help elucidate the mechanisms underlying the therapeutic effects of OA treatments.

In this study, we aimed to evaluate the therapeutic efficacy of NWG11-02 in a monosodium iodoacetate (MIA)-induced OA rat model. The results demonstrated significant joint-protective, anti-inflammatory, and cartilage-preserving effects. The treatment downregulated catabolic markers such as COMP, CTX-II, MMP-3 and MMP-13, while upregulating anabolic markers including ACAN and COL2A1. In addition, pro-inflammatory mediators including iNOS, COX-2, TNF-α, IL-1β, and IL-6 were significantly suppressed. These results suggest that NWG11-02 exerts therapeutic effects by modulating cartilage metabolism and inflammatory responses.

## Materials and Methods

### Preparation of OE, SJFE and NWG11-02

Oleaster extract (OE), SJFE and NWG11-02 were provided by NOVAWells Co., Ltd. (Republic of Korea). For the preparation of the OE, whole fruits were extracted with 50% ethanol (v/v) at 70 ±5°C for 4±1h using a solvent-to-solid ratio of 10:1 (w/v). The crude extract was filtered through a polyethylene (PE) cartridge filter, concentrated under reduced pressure using a rotary evaporator, and subsequently spray-dried to obtain the powdered extract. SJFE was imported by NOVAWells Co., Ltd. from GreenChem (Bengaluru, India; Lot No. REX/ 24016) and prepared by extracting the fruits with 60% ethanol (v/v) at 80 ± 5°C for 4 ± 1 h. The extract was vacuum-concentrated, cooled, and subjected to low-temperature precipitation for 16–24 h. After decanting approximately one-third of the supernatant, the remaining concentrate was spray-dried to yield the powdered extract. SJFE was a standardized extract containing more than 10% sophoricoside, as determined by HPLC analysis. For the preparation of the OE–SJFE mixture, a concentrated extract of Oleaster is mixed with SJFE at a ratio of 3:1 (w/w). The total mixture of Oleaster extract and SJFE was blended with maltodextrin at a 1:1 (w/w) ratio and subsequently spray-dried to obtain the final powdered complex, designated as NWG11-02. The mixing ratio of 3:1 was determined based on internal optimization studies (unpublished data), which identified this formulation as the most effective combination for joint and cartilage health. The optimal ratio and composition have been filed for patent protection (Korean Patent Application No. 10-2024-0181894: *Extract Complex of Oleaster Fruit and Sophora Japonica Fruit, Food and Health Functional Food Containing the Same for Improving Joint and Cartilage Health*).

### Reagents

MIA, celecoxib (CLX), and toluidine blue were purchased from Sigma-Aldrich (USA). The Hematoxylin and Eosin (H&E) staining kit was obtained from Abcam (UK).

### Animal Experiments

Six-week-old male Sprague–Dawley rats were obtained from Orient Bio (Republic of Korea) and acclimated for one week under standard laboratory conditions (22 ± 2°C, 50 ± 10% humidity, 12 h light/dark cycle) with *ad libitum* access to food and water. All experimental procedures were approved by the Institutional Animal Care and Use Committee (IACUC) of Sejong University (Approval No. IACUC2404-005) and conducted in accordance with institutional and national ethical guidelines.

### Induction of OA and Sample Administration

After the acclimation period, OA was induced in the right knee joint of rats *via* a single intra-articular injection of MIA, except the normal control group. A total of eight experimental groups (*n* = 9 per group) were established: normal control, MIA-induced group, CLX (3 mg/kg), OE (40 mg/kg), SJFE (40 mg/kg), and NWG11-02-treated groups at 40, 80, and 120 mg/kg. All treatments were administered orally once daily for 4 weeks. Throughout the experimental period, the health condition of the animals was monitored daily, and body weight was measured once per week.

### Weight-Bearing Index Measurement

Weight-bearing index (WBI) was measured using the Librae Incapacitance Tester for Rats (Model No. C10SSMTV2026P; Ugo Basile, Italy) at baseline (prior to MIA injection) and weekly thereafter for 4 weeks (days 7, 14, 21, and 28). Rats were placed in a transparent acrylic chamber with their hind limbs extended separately onto two force sensors, allowing the distribution of weight on each hind limb to be recorded. Each measurement was repeated three times for 15 s, and the average value was used. WBI was calculated as the percentage of weight supported by the right hind limb relative to the total weight supported by both limbs.

### Histological Analysis

Rat knee joint tissues were fixed in 10% neutral-buffered formalin, embedded in paraffin, and sectioned at a thickness of 3–5 μm. The sections were deparaffinized with xylene and rehydrated through a graded ethanol series. For general histological assessment, sections were stained with hematoxylin and eosin (H&E) for 5 min. For proteoglycan visualization, adjacent sections were stained with toluidine blue for 5–10 min. Images of stained sections were acquired using the Leica THUNDER Imaging System (Leica Microsystems, Germany) at the Biopolymer Research Center for Advanced Materials (BRCAM). Histological features of articular cartilage were semi-quantitatively evaluated using the Mankin scoring system, based on cartilage structure, cellularity, matrix staining intensity, and tidemark integrity.

### Serum Analysis

After CO_2_ anesthesia, blood was collected from the abdominal aorta of rats. The collected blood was incubated at room temperature for 30 min and centrifuged at 1,000 ×*g* for 15 min to obtain serum. Cartilage degradation markers COMP and CTX-II were quantified using ELISA kits (MyBioSource, USA) according to the manufacturer’s protocols. Hepatic injury markers (ALT, AST, and ALP) and the renal function marker (BUN, Creatinine) were analyzed at T&P Bio (Republic of Korea) using the BC-5000 Vet Auto Hematology Analyzer (Mindray, China).

### Quantitative Real-Time Polymerase Chain Reaction

Total RNA was extracted from rat knee cartilage tissues using TRIzol reagent (Thermo Fisher Scientific, USA) and quantified using a NanoDrop OneC Microvolume UV-Vis spectrophotometer (Thermo Fisher Scientific). Complementary DNA (cDNA) was synthesized from 1 μg of RNA using the amfiRivert cDNA Synthesis Platinum Master Mix (GenDEPOT, USA). RT-qPCR was conducted with 2× GreenStar qPCR Master Mix (Bioneer, Republic of Korea). Primer sequences are listed in Table 1, and GAPDH was used as the internal control. Relative gene expression levels were calculated using the 2^–ΔΔCt^ method.

### Western Blotting

Total protein was extracted from rat knee cartilage tissues using lysis buffer (Cell Signaling Technology, USA). Protein concentration was determined using the Pierce BCA Protein Assay Kit (Thermo Fisher Scientific). Equal amounts of protein were separated via 10% SDS-PAGE and transferred onto PVDF membranes. Membranes were blocked with 5% bovine serum albumin (BSA) and incubated overnight at 4°C with the following primary antibodies: COX-2 (Santa Cruz Biotechnology, sc-19999), TNF-α (Abcam, ab307164), IL-1β (Invitrogen, P420B), IL-6 (Abcam, ab208113), MMP-3 (Invitrogen, PA5-119639), MMP-13 (Biorbyt, orb214263), and Aggrecan (Invitrogen, MA3-16888). After incubation with HRP-conjugated secondary antibodies, protein bands were visualized using a chemiluminescence detection system (LuminoGraph III Lite; ATTO, Japan) at the Biopolymer Research Center for Advanced Materials (BRCAM). Band intensities were quantified using ImageJ software (NIH, USA).

### Statistical Analysis

All experiments were performed at least in triplicate, and data are presented as the mean ± standard deviation. Statistical analysis was conducted using one-way ANOVA followed by Dunnett’s post hoc test. Statistical significance relative to the control group was indicated by ‘#’, and significance relative to the MIA group by ‘*’. A *p*-value of < 0.05 was considered statistically significant.

## Results

### OE, SJFE, and NWG11-02 Improve WBI and Cartilage Integrity in Rats with Osteoarthritis

**Fig. 1:**
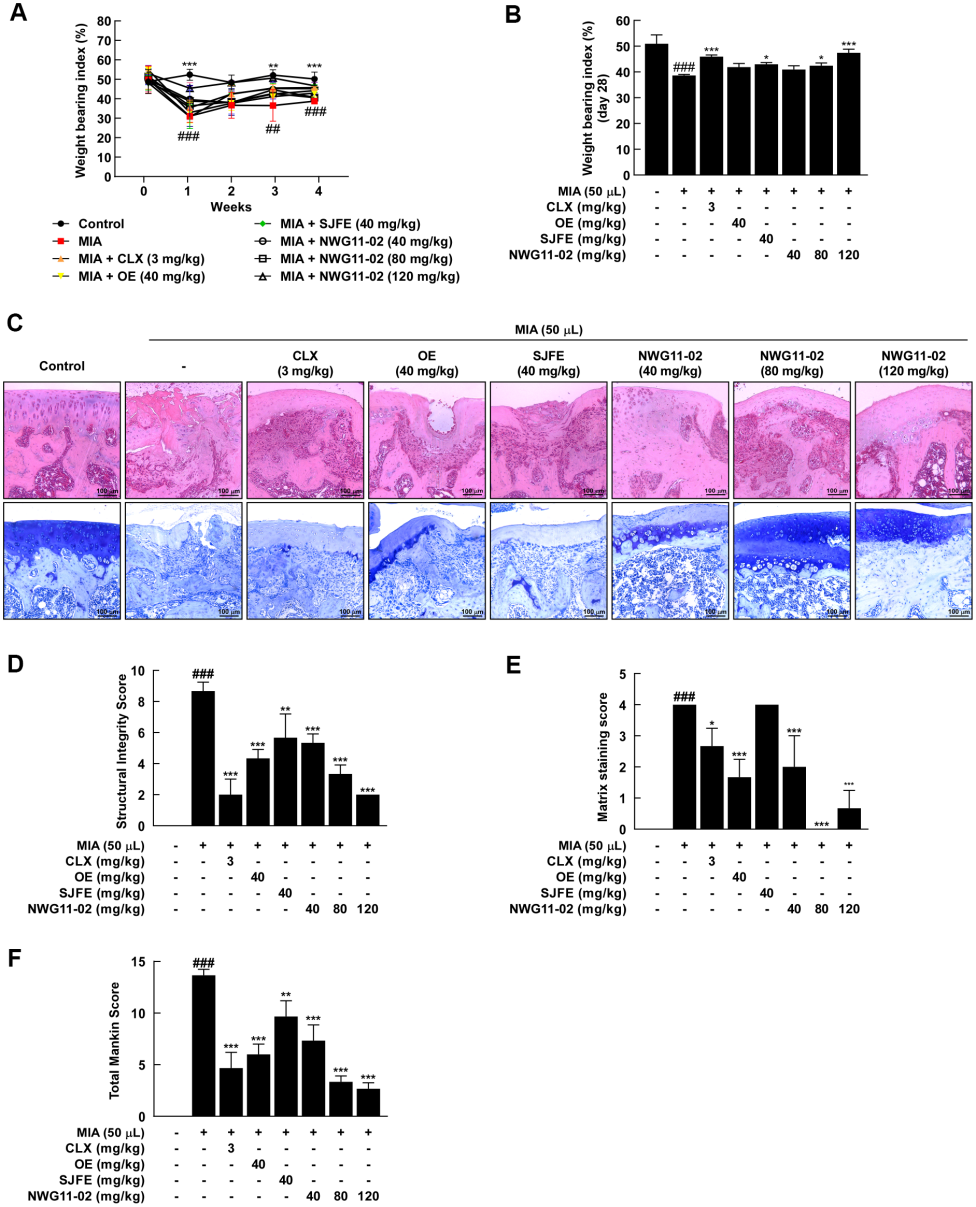
The Protective effects of OE, SJFE, and NWG11-02 on hind-limb function and cartilage degeneration in MIA-induced OA rats. (**A**, **B**) Weight-bearing index (WBI, %) measured weekly and on day 28 to evaluate hind-limb function. (**C**) Representative histological images of knee joints stained with hematoxylin and eosin (H&E) and toluidine blue. (**D**) Structural integrity scores based on H&E staining, reflecting cartilage surface structure (0–6) and chondrocyte distribution (0–3), assessed as components of the Mankin grading system. (**E**) Matrix staining scores based on toluidine blue staining, evaluated according to Mankin criteria (0 = strong staining; 4 = no staining), reflecting proteoglycan content in the cartilage matrix. (**F**) Total Mankin histological scores calculated by summing the values from (**D, E**), and tidemark integrity scores, representing overall severity of cartilage degeneration. Data are presented as mean ± SD. ^#^*p* < 0.05, ^##^*p* < 0.01, ^###^*p* < 0.001 vs. control; *p* < 0.05, *p* < 0.01, *p* < 0.001 vs. MIA.

WBI, which reflects joint pain and functional impairment in OA models, was measured weekly to evaluate therapeutic recovery of joint function. One week after OA induction, WBI significantly decreased compared to normal control, indicating successful development of joint impairment (Fig. 1A). From week 3, rats treated with the NWG11-02 (120 mg/kg) exhibited marked functional recovery. By week 4, functional improvement was also observed in the CLX group, as well as in the SJFE and NWG11-02 groups at both 80 and 120 mg/kg (Fig. 1B). Histological evaluation of knee joints was performed using H&E and toluidine blue staining. Severe cartilage degradation and proteoglycan loss were observed in OA-induced joints (Fig. 1C). Histological analysis using H&E staining revealed that the MIA-induced OA group exhibited irregularities in the cartilage surface and disrupted chondrocyte organization. In contrast, the sample-treated group demonstrated improved cartilage architecture, characterized by a smoother surface and more organized chondrocyte alignment. Toluidine blue staining showed a significant depletion of proteoglycans in the OA group, whereas the treated group exhibited more intense blue staining, reflecting a higher level of preserved extracellular matrix components. The MIA group exhibited elevated scores for both structural integrity and matrix staining, reflecting pronounced cartilage degradation and reduced proteoglycan levels. These scores were significantly decreased following treatment with OE, SJFE, or NWG11-02. NWG11-02 group maintained relatively intact cartilage architecture and matrix composition (Fig. 1D-1E). Similarly, the total Mankin score, calculated from structural, matrix, and tidemark evaluations, was notably reduced in all treatment groups, with the combined treatment showing the greatest protective effect (Fig. 1F). These results indicate that NWG11-02 extract effectively alleviates OA-associated functional impairment and structural cartilage damage.

### NWG11-02 Attenuates Cartilage Degradation and Promotes Cartilage Matrix Synthesis

**Fig. 2:**
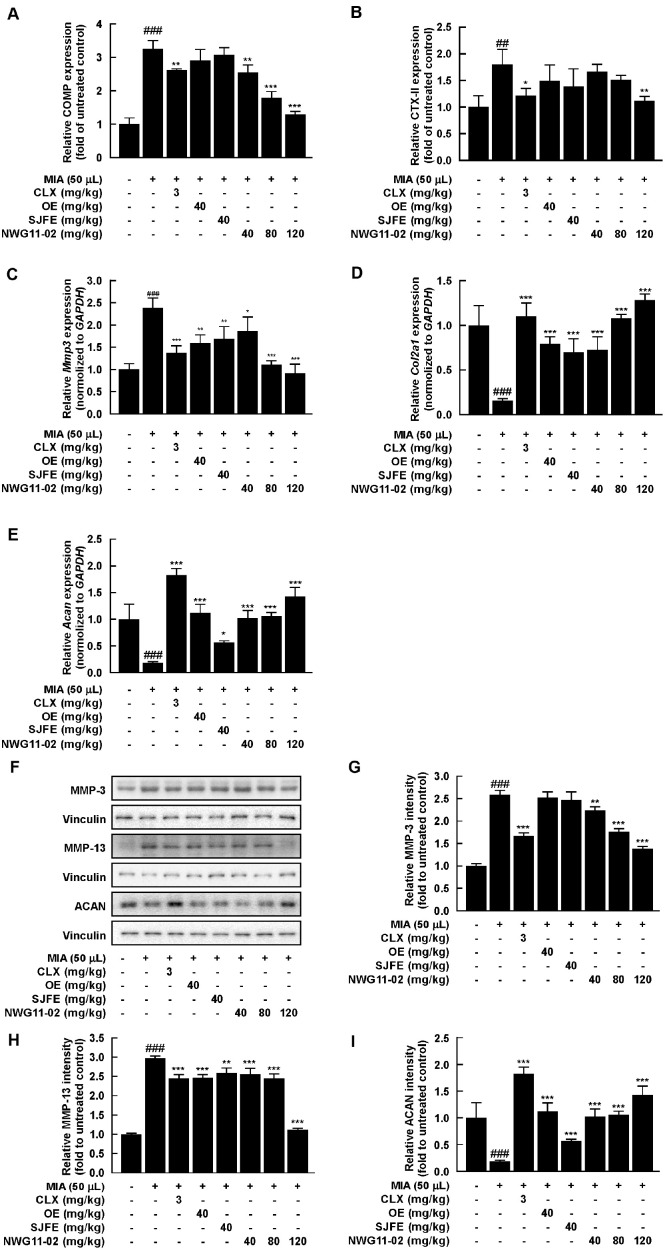
Modulatory effects of OE, SJFE, and NWG11-02 on cartilage matrix degradation and synthesis in MIA-induced OA rats. (**A, B**) Serum levels of COMP and CTX-II measured using ELISA. (**C–E**) Relative mRNA expression of MMP-3, COL2A1 and ACAN determined by qRT-PCR. (**F-I**) Protein expression levels of MMP-3, MMP-13, and ACAN assessed by Western blotting. ^#^*p* < 0.05, ^##^*p* < 0.01, ^###^*p* < 0.001 vs. control; *p < 0.05, **p < 0.01, ***p < 0.001 vs. MIA.

To investigate the effects of NWG11-02 on cartilage metabolism, representative markers of cartilage degradation and synthesis were analyzed in joint tissues. MIA treatment led to increased levels of catabolic markers and reduced expression of cartilage matrix components, while CLX treatment reversed these changes to a significant extent. ELISA results showed that cartilage oligomeric matrix protein (COMP), a structural component released during cartilage breakdown, and C-terminal telopeptide of type II collagen (CTX-II), a collagen degradation product, were effectively reduced by NWG11-02 treatment (Fig. 2A and 2B). The mRNA expression of MMP-3 was also suppressed following administration of NWG11-02 extract (Fig. 2C), whereas the mRNA expression of COL2A1 and ACAN were increased (Fig. 2D and 2E). Protein expression levels of MMP-3, MMP-13, and ACAN were assessed by western blot (Fig. 2F). Densitometric analysis (Fig. 2G-2I) confirmed that NWG11-02 extract restored all three markers toward control levels. Notably, the high-dose group exhibited expression patterns comparable to those of the normal group. These findings indicate that NWG11-02 extract not only inhibits cartilage degradation but also promotes matrix regeneration, contributing to overall cartilage protection.

### NWG11-02 Suppresses Pro-Inflammatory Markers in Joint Tissue

**Fig. 3:**
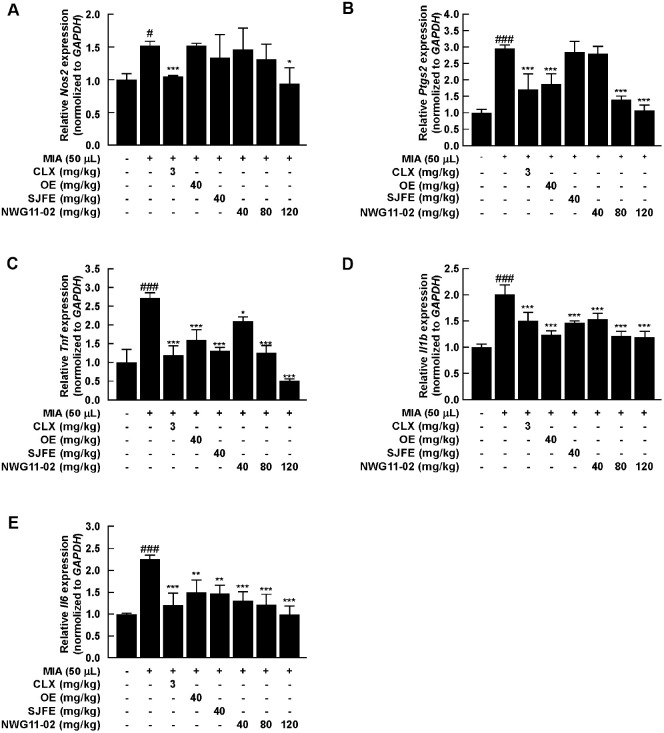
Inhibitory effects of OE, SJFE, and NWG11-02 on inflammatory gene expression in the knee joint tissues of MIA-induced OA rats. (**A–E**) Relative mRNA expression levels of iNOS, COX-2, TNF-α, IL-1β and IL-6 determined by qRT-PCR. ^#^*p* < 0.05, ^##^*p* < 0.01, ^###^*p* < 0.001 vs. control; *p < 0.05, **p < 0.01, ***p < 0.001 vs. MIA.

**Fig. 4:**
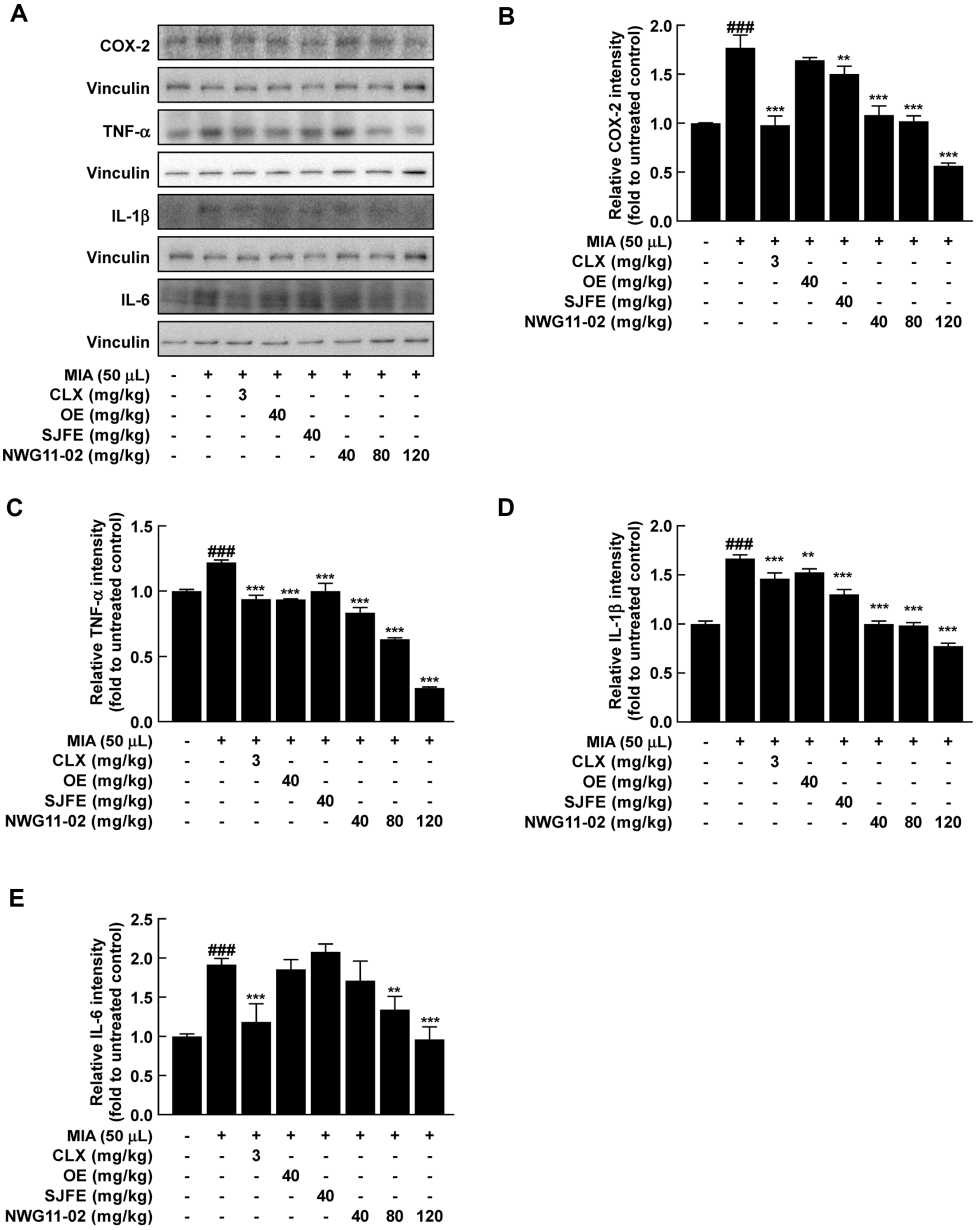
Inhibitory effects of OE, SJFE, and NWG11-02 on inflammatory protein expression in the knee joint tissues of MIA-induced OA rats. (**A–E**) Protein expression of COX-2, TNF-α, IL-1β, and IL-6 analyzed by western blot. Data are presented as mean ± SD. ^#^*p* < 0.05, ^##^*p* < 0.01, ^###^*p* < 0.001 vs. control; *p < 0.05, **p < 0.01, ***p < 0.001 vs. MIA.

To evaluate the anti-inflammatory effects of the mixture extract, mRNA and protein expression levels of representative pro-inflammatory markers were analyzed in joint tissues following 4 weeks of oral administration. RT-qPCR analysis showed that the mRNA expression of Nos2 (a gene encoding iNOS) and Ptgs2 (a gene encoding COX-2) was markedly upregulated by MIA-induced joint inflammation. These increases were significantly reduced in the CLX-treated group. Notably, NWG11-02 treatment led to a clear dose-dependent downregulation of both genes (Fig. 3A and 3B). A similar pattern was observed for the inflammatory cytokines. Their mRNA expression levels (TNF-α, IL-1β, and IL-6) decreased progressively with increasing doses of NWG11-02, supporting its anti-inflammatory potential at the transcriptional level (Fig. 3C-3E). Protein expression was evaluated by western blot (Fig. 4A). MIA induction resulted in elevated levels of COX-2, TNF-α, IL-1β, and IL-6, whereas these increases were effectively suppressed by CLX. NWG11-02 also reduced protein expression of these markers in a dose-dependent manner. Densitometric analysis confirmed that band intensities were notably lower in NWG11-02 groups than in those treated with single extracts (Fig. 4B-4E). These results suggest that NWG11-02 extract exerts strong anti-inflammatory effects by suppressing key inflammatory mediators at both gene and protein levels.

### Oral Administration of OE, SJFE, and NWG11-02 Does Not Induce Toxicity in Sprague–Dawley Rats

**Fig. 5:**
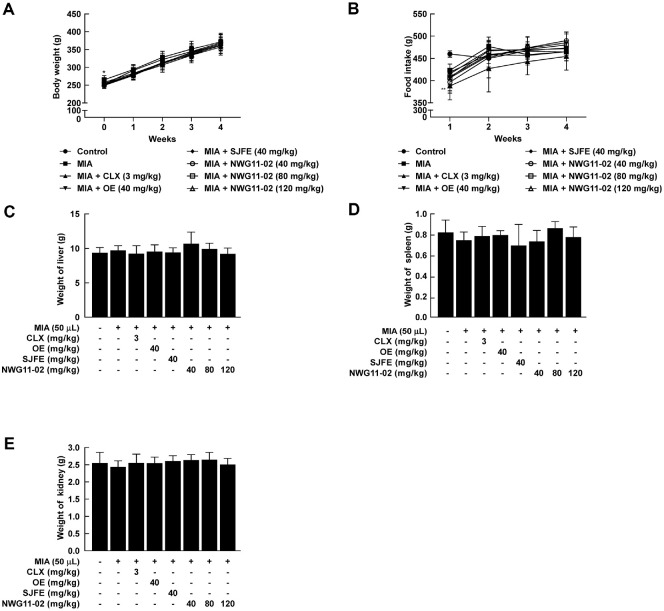
Effects of OE, SJFE, and NWG11-02 on food intake, body weight, and organ weights in MIA-induced OA rats. (**A**) Weekly body weight (g) and (**B**) food intake (g) during the 4-week oral administration of OE, SJFE, or NWG11- 02 (40, 80, or 120 mg/kg). (**C–E**) Organ weights of the (**C**) liver, (**D**) spleen, and (**E**) kidney (g), measured at the time of sacrifice. CLX (3 mg/kg) was used as a positive control. Data are presented as mean ± SD (*n* = 4). *p < 0.05, **p < 0.01 vs. control.

**Fig. 6:**
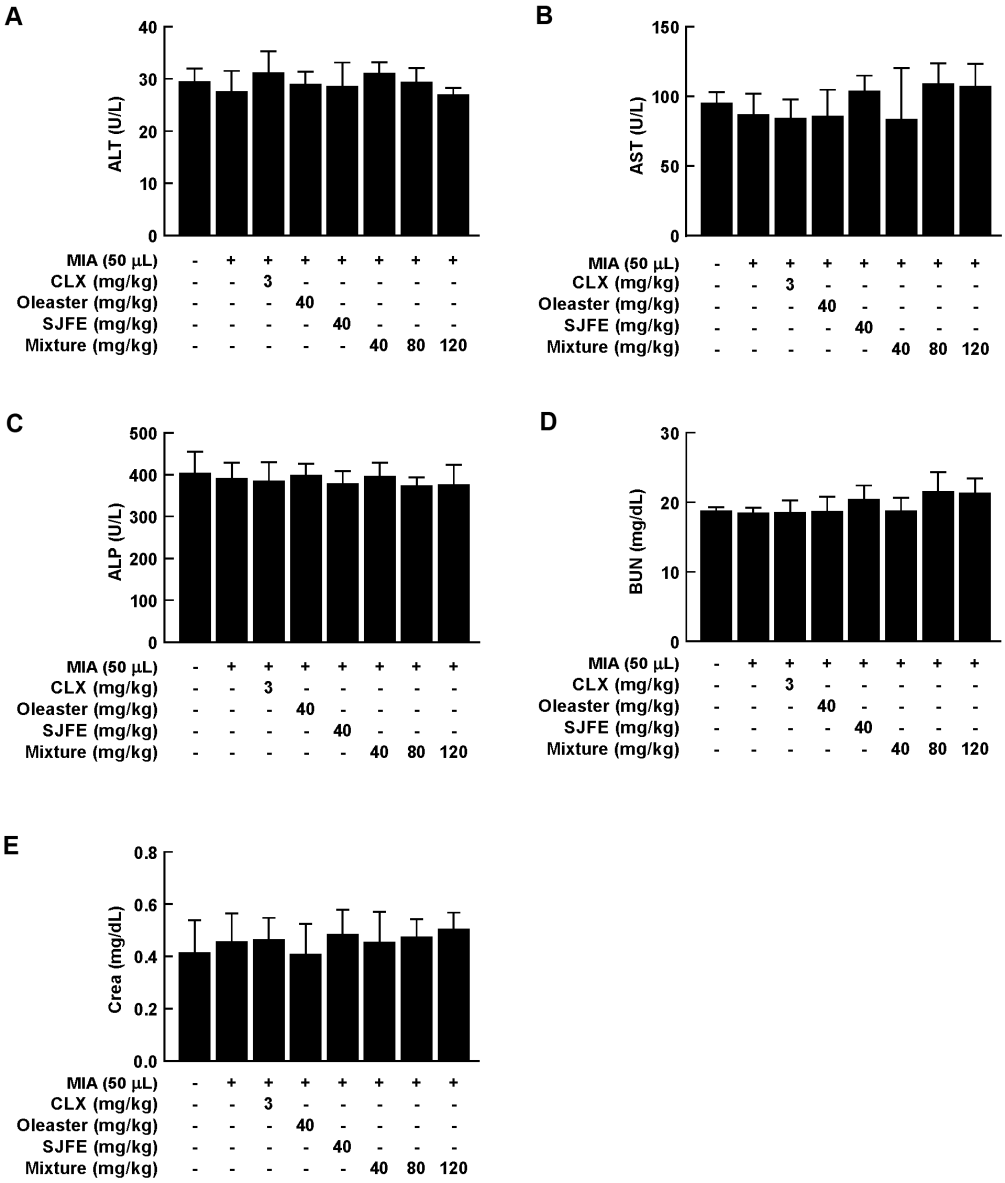
Effects of OE, SJFE, and NWG11-02 on serum biochemical parameters in MIA-induced OA rats. (**A–E**) Serum levels of (**A**) ALT (U/L), (**B**) AST (U/L), (**C**) ALP (U/L), (**D**) BUN (mg/dL), and (**E**) Creatinine (mg/dL) were measured to evaluate hepatic and renal function after 4 weeks of treatment. Data are presented as mean ± SD (*n* = 4). *p < 0.05, **p < 0.01 vs. control.

To assess *in vivo* toxicity, OE, SJFE, or NWG11-02 was orally administered to Sprague–Dawley rats. Body weight, food intake, and organ weights were monitored throughout the experimental period. Body weight remained consistent across all groups, showing no appreciable deviation from control levels (Fig. 5A). Similarly, food intake was maintained without marked variation (Fig. 5B). At the end of the study, liver, spleen, and kidney weights were comparable among groups, indicating that the treatments did not elicit systemic toxicity (Fig. 5C-5E). These findings suggest that NWG11-02 extract can be safely administered without inducing adverse physiological effects. To assess potential liver and kidney toxicity, serum levels of alanine aminotransferase (ALT), aspartate aminotransferase (AST), and alkaline phosphatase (ALP) were examined. None of the treatment groups showed significant increases in these enzymes, indicating no hepatic injury (Fig. 6A-6C). Similarly, blood urea nitrogen (BUN) and creatinine (Crea) levels remained stable across all groups, suggesting that renal function was not adversely affected (Fig. 6D-6E). Overall, these findings indicate that OE, SJFE, and NWG11-02 can be administered orally without causing systemic or organ-specific toxicity at the tested doses.

## Discussion

In this study, we evaluated the therapeutic potential of OE, SJFE, and NWG11-02 in a MIA-induced OA rat model. MIA, a glycolysis inhibitor, selectively induces chondrocyte death and cartilage degradation, and is widely used to establish OA in preclinical studies [26, 27]. CLX, a selective COX-2 inhibitor, was used as a positive control and served as a reference for evaluating the efficacy of the test materials. All treatment groups showed varying degrees of improvement in joint function and cartilage preservation.

Notably, the medium-dose of NWG11-02 (80 mg/kg) exhibited greater efficacy than either OE or SJFE alone (40 mg/kg) across various evaluated parameters, including multiple outcomes. Improvements in weight-bearing function at week 4 were more pronounced in NWG11-02 group (Fig. 1B), and histological assessments revealed better preservation of cartilage structure, with lower Mankin scores and more intense toluidine blue staining (Fig. 1C and 1D). At the molecular level, NWG11-02 more effectively modulated inflammatory and catabolic responses, with favorable trends observed in several key markers related to joint degradation and matrix integrity. Importantly, many of these improvements appeared to follow a dose-dependent pattern, with the high-dose of NWG11-02 (120 mg/kg) producing the most consistent effects. These findings suggest that NWG11-02 may offer broader therapeutic coverage than either extract alone, even under equivalent-dose conditions. In particular, the mixture not only suppressed pro-inflammatory cytokines (*e.g.*, TNF-α, IL-1β, IL-6) (Figs. 3 and 4) and matrix-degrading enzymes (*e.g.*, MMP-3) but also helped restore cartilage matrix components such as COL2A1 and ACAN. These dual effects on catabolic suppression and anabolic support highlight the comprehensive joint-protective potential of NWG11-02.

The enhanced efficacy observed in NWG11-02 group may be attributed to complementary interactions between the bioactive compounds present in OE and SJFE. Both extracts are rich in flavonoids such as kaempferol, quercetin, and rutin, which have been reported to exert anti-inflammatory, antioxidant, and cartilage-protective effects [28, 29]. For example, kaempferol has been shown to inhibit COX-2 and TNF-α expression and reduce oxidative stress in OA models [30], while flavonoids derived from SJFE have been linked to the regulation of inflammatory signaling and suppression of matrix-degrading enzymes [31]. Although the present study did not directly investigate signaling pathways, the combination of these phytochemicals in the mixture may act through converging mechanisms to modulate key aspects of OA pathology. This could partly explain the improved functional and histological outcomes observed in NWG11-02 groups, even under equivalent or moderate-dose conditions. These observations support the rationale for using well-characterized botanical mixtures to enhance the therapeutic spectrum of natural OA treatments.

While the present study provides supportive evidence for the therapeutic potential of NWG11-02 in alleviating OA-related pathological features, several limitations should be considered. Although improvements in joint function, cartilage integrity, and molecular marker expression were observed, the study did not directly investigate intracellular signaling pathways such as NF-κB or MAPK, which are critically involved in OA progression [32]. Furthermore, as the experimental model was limited to MIA-induced OA in rats, additional studies—including long-term evaluations and clinical trials—will be necessary to establish translational relevance. Nonetheless, NWG11-02 demonstrated greater overall efficacy than either extract alone, suggesting potential synergistic interactions and highlighting the value of combining well-characterized botanical agents as a multi-targeted strategy for OA management.

Taken together, these findings support the potential utility of the NWG11-02 as a natural intervention for OA. By targeting multiple aspects of joint pathology, NWG11-02 may provide a broader therapeutic benefit than either extract alone and warrants further investigation in both mechanistic and clinical settings.

## Figures and Tables

**Table 1:** Sequences of PCR-Primers.

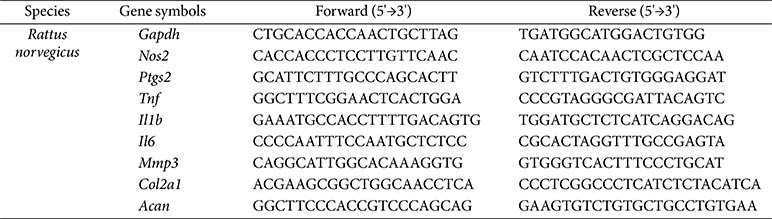

## References

[1] Neogi T (2013). The epidemiology and impact of pain in osteoarthritis. Osteoarthritis Cartilage.

[2] He Y, Li Z, Alexander PG, Ocasio-Nieves BD, Yocum L, Lin H (2020). Pathogenesis of osteoarthritis: risk factors, regulatory pathways in chondrocytes, and experimental models. Biology (Basel).

[3] Li GY, Yin JM, Gao JJ, Cheng TS, Pavlos NJ, Zhang CQ (2013). Subchondral bone in osteoarthritis: insight into risk factors and microstructural changes. Arthritis Res. Ther..

[4] Wojcieszek A, Kurowska A, Majda A, Liszka H, Gadek A (2022). The impact of chronic pain, stiffness and difficulties in performing daily activities on the quality of life of older patients with knee osteoarthritis. Int. J. Environ. Res. Public Health.

[5] Cui A, Li H, Wang D, Zhong J, Chen Y, Lu H (2020). Global, regional prevalence, incidence and risk factors of knee osteoarthritis in population-based studies. EClinicalMedicine.

[6] Long H, Liu Q, Yin H, Wang K, Diao N, Zhang Y (2022). Prevalence trends of site-specific osteoarthritis from 1990 to 2019: findings from the global burden of disease study 2019. Arthritis Rheumatol..

[7] Bannwarth B (2006). Acetaminophen or NSAIDs for the treatment of osteoarthritis. Best Pract. Res. Clin. Rheumatol..

[8] Lee SH, Seo HY, Kim HR, Song EK, Seon JK (2021). Older age increases the risk of revision and perioperative complications after high tibial osteotomy for unicompartmental knee osteoarthritis. Sci. Rep..

[9] Hamidpour R, Hamidpour S, Hamidpour M, Shahlari M, Sohraby M, Shahlari N, *et al*. 2017. Russian olive (*Elaeagnus angustifolia* L.): from a variety of traditional medicinal applications to its novel roles as active antioxidant, anti-inflammatory, anti-mutagenic and analgesic agent. *J. Tradit. Complement. Med.* **7:** 24-29. 10.1016/j.jtcme.2015.09.004 28053884 PMC5198788

[10] Li H, Yang J, Deng W, Zhou T, Guo D, Li Y (2025). Network pharmacology and in-depth blood proteomics reveal the mechanism of Buqi Tongluo capsules in treating bone destruction in osteoarthritis. Phytomedicine.

[11] Lou C, Lin C, Wang W, Jiang H, Cai T, Lin S (2023). Extracts of *Oldenlandia diffusa* protects chondrocytes via inhibiting apoptosis and associated inflammatory response in osteoarthritis. J. Ethnopharmacol..

[12] Zeng Y, Yu S, Lu L, Zhang J, Xu C (2024). Ginger-derived nanovesicles attenuate osteoarthritis progression by inhibiting oxidative stress via the Nrf2 pathway. Nanomedicine (Lond).

[13] Panahi Y, Alishiri GH, Bayat N, Hosseini SM, Sahebkar A (2016). Efficacy of elaeagnus *Angustifolia* extract in the treatment of knee osteoarthritis: a randomized controlled trial. EXCLI J..

[14] Mofid M, Sadraie SH, Imani H, Torkaman G, Kaka G, Naghii MR (2020). The effect of elaeagnus angustifolia extract on the joint friction and antioxidant activity in knee non-traumatic osteoarthritis model in rat. MLTJ-Muscles Ligaments Tendons. J..

[15] Amir Akbarnejad Eshkalak LK (2022). Evaluation of alcoholic extract of *Elaeagnus Angustifolia* L. in diminishing proinflammatory genes in a model of CA-II-induced OA mice. Int. J. Clin. Exp. Med. Sci..

[16] Chakuleska L, Shkondrov A, Popov G, Zlateva-Panayotova N, Petrova R, Atanasova M (2022). Beneficial effects of the fructus Sophorae extract on experimentally induced osteoporosis in New Zealand white rabbits. Acta Pharm..

[17] Afshar F, Abdolahi N, Amin G, Esmaily H, Ziayie S, Azimi S (2022). A randomized, double-blind placebo-controlled phase I clinical study on safety and efficacy of the G-Rup(R) syrup (a mixture of ginger extract and honey) in symptomatic treatment of knee osteoarthritis. J. Clin. Pharm. Ther..

[18] Choi YH, Kang HJ (2016). Fructus sophorae attenuates secretion of proinflammatory mediators and cytokines through the modulation of NF-kappaB and MAPK signaling pathways in LPS-stimulated RAW 264.7 macrophages. Gen. Physiol. Biophys..

[19] Han HM, Hong SH, Park HS, Jung JC, Kim JS, Lee YT (2017). Protective effects of *Fructus sophorae* extract on collagen-induced arthritis in BALB/c mice. Exp. Ther. Med..

[20] Lee WJ, Kim KM, Lee S, Park SY, Kim H, Imm JY (2024). Alleviating effect of a flower extract of *Styphnolobium japonicum* L. on symptoms of experimentally induced osteoarthritis in rats. Appl. Sci..

[21] Vaidya N, Agarwal R, Dipankar DG, Patkar H, Ganu G, Nagore D, *et al*. 2025. Efficacy and safety of boswellia serrata and *Apium graveolens* L. extract against knee osteoarthritis and cartilage degeneration: a randomized, double-blind, multicenter, placebocontrolled clinical trial. *Pharm. Res.* **42:** 249-269. 10.1007/s11095-025-03818-2 39875757 PMC11880083

[22] Schuerwegh AJ, Dombrecht EJ, Stevens WJ, Van Offel JF, Bridts CH, De Clerck LS (2003). Influence of pro-inflammatory (IL-1α, IL-6, TNF-α, IFN-γ) and anti-inflammatory (IL-4) cytokines on chondrocyte function. Osteoarthr. Cartil..

[23] Tu M, Yang M, Yu N, Zhen G, Wan M, Liu W (2019). Inhibition of cyclooxygenase-2 activity in subchondral bone modifies a subtype of osteoarthritis. Bone Res..

[24] Mukherjee A, Das B (2024). The role of inflammatory mediators and matrix metalloproteinases (MMPs) in the progression of osteoarthritis. Biomater. Biosyst..

[25] Ouyang Z, Dong L, Yao F, Wang K, Chen Y, Li S (2023). Cartilage-related collagens in osteoarthritis and rheumatoid arthritis: from pathogenesis to therapeutics. Int. J. Mol. Sci..

[26] Maouche A, Boumediene K, Bauge C (2024). Bioactive compounds in osteoarthritis: molecular mechanisms and therapeutic roles. Int. J. Mol. Sci..

[27] Pitcher T, Sousa-Valente J, Malcangio M (2016). The monoiodoacetate model of osteoarthritis pain in the mouse. J. Vis. Exp..

[28] Heydari Nasrabadi M, Parsivand M, Mohammadi N, Asghari Moghaddam N (2022). Comparison of *Elaeagnus angustifolia* L. extract and quercetin on mouse model of knee osteoarthritis. J. Ayurveda Integr. Med..

[29] Liu T, Su B (2021). *Styphnolobium japonicum* (L.) schott flower extract alleviates oxidative stress and inflammatory factors in the adjuvant-induced arthritis rat model. J. Pain Res..

[30] Hussain MS, Altamimi ASA, Afzal M, Almalki WH, Kazmi I, Alzarea SI (2024). Kaempferol: paving the path for advanced treatments in aging-related diseases. Exp. Gerontol..

[31] Yeom M, Ji H, Shin J, Cho E, Ryu DH, Park D (2022). The alleviating effect of *Lagerstroemia indica* flower extract on stretch marks through regulation of mast cells. Molecules.

[32] Zhu L, Bi Y, Liang T, Zhang P, Xiao X, Yu T (2025). Ginkgetin delays the progression of osteoarthritis by inhibiting the NF-kappaB and MAPK signaling pathways. J. Orthop. Surg. Res..

